# Phenotypic Plasticity in Juvenile Frogs That Experienced Predation Pressure as Tadpoles Does Not Alter Their Locomotory Performance

**DOI:** 10.3390/biology12030341

**Published:** 2023-02-21

**Authors:** Junkyu Park, Yuno Do

**Affiliations:** Department of Biological Science, Kongju National University, Gongju 32588, Republic of Korea

**Keywords:** adaptive traits, anatomical morphology, locomotor performance, non-consumption effect, phenotype plasticity

## Abstract

**Simple Summary:**

In this study, we investigated whether predation pressures in the early life history of frogs can result in an anuran-affect behavior response in the later stages of their life history. Tadpoles that undergo predation pressure possess smaller body sizes, slower development rates, and broader caudal fins. In particular, juvenile frogs that undergo predation pressure from the tadpole stage also metamorphosize later, develop longer limbs, narrower skulls, and demonstrate other finer changes in the skeleton of the lower body. On this note, we predicted that this would change the locomotory performance of such juvenile frogs in terms of their swimming and jumping speed, but such an eventuality did not take place. It appears, then, that predation pressure in the early life history of frogs does not alter their jumping and swimming speed abilities. Moreover, such a fact suggests that this morphological change may not be a predator-adaptive response.

**Abstract:**

Anuran species can respond to environmental changes via phenotypic plasticity, which can also result in ecological impacts across the life history of such species. We investigated the effects of predation pressure (i.e., the non-consumption effect) from the dragonfly larva (*Anax parthenope*) on the phenotypical change of tadpoles into juvenile frogs (specifically the black-spotted pond frog, *Pelophylax nigromaculatus*), and also analyzed the impact of morphological changes on locomotory performance after metamorphosis. The experiments on predator impact were conducted in the laboratory. Body length, weight, development timing, and metamorphosis timing in the presence of dragonfly nymphs were measured in both tadpoles and juvenile frogs. The body and tail shapes of the tadpoles, as well as the skeletal shape of the juvenile frogs, were analyzed using landmark-based geometric morphometrics. Furthermore, the locomotory performance of the juvenile frogs was tested by measuring their jumping and swimming speeds. Tadpoles that had grown with predators possessed smaller bodies, deeper tail fins, and slower development rates, and they waited longer periods of time before commencing metamorphosis. Having said this, however, the effect of predator cues on the body length and weight of juvenile frogs was not found to be significant. These juvenile frogs possessed longer limbs and narrower skulls, with subtle morphological changes in the pelvis and ilium, but there was no subsequent difference in their swimming and jumping speeds. Our results showed that the changes in anatomical traits that can affect locomotor performance are so subtle that they do not affect the jumping or swimming speeds. Therefore, we support the view that these morphological changes are thus by-products of an altered tadpole period, rather than an adaptive response to predator-escape ability or to post-metamorphosis life history. On the other hand, delayed metamorphosis, without an increase in body size, may still be disadvantageous to the reproduction, growth, and survival of frogs in their life history following metamorphosis.

## 1. Introduction

Phenotypic plasticity is the ability of an organism to produce a suitable phenotype in response to the environmental variability that is present in their habitats; furthermore, such a response can influence the survival or reproduction of a species [1]. Among the many animal groups, anurans especially exhibit an adaptive plasticity via their response to various types of habitats and their complex life histories [2,3,4,5,6,7,8,9]. A phenomenon that is representative of such phenotypic plasticity in anurans is the change in tail shape of tadpoles who are exposed to predators. Tadpoles that are exposed to predators have been found to possess deeper tail fins [4,7,10,11]. The change in the tail fin in response to predators occurs through the mediation of the glucocorticoid hormone, corticosterone [12]. Morphological changes in the tail may vary depending on the species or hunting strategy of the predator [13]. The deepened tail fin provides a lure effect, thereby increasing the survival rate of tadpoles against predators [10].

Phenotypic plasticity does not occur within only one stage of the life cycle. A plastic response can be induced and combined at multiple ontogenetic stages of development; in addition, it can also result in either a temporary or lasting effect. For example, the loss of tail tissue in tadpoles can lead to smaller frogs after their metamorphosis [14]. Additionally, the predators or competitors who caused such a response play a part in the variation of shape of not only the tadpole, but also the juvenile frogs after metamorphosis. This is evidenced by the fact that the changed shapes did not converge [6,15]. The plastic responses may be more complicated due to the fact that, unlike other vertebrates, most anurans experience metamorphosis from the tadpole to adult frog stage. In toads, who can defend against predators using toxic chemicals, predation pressure at the tadpole stage alters the investment into toxic chemical defenses, which can serve as defenses after the process of metamorphosis [16]. Additionally, there was a study regarding the physiological effect in respect to the lasting effect of a group demonstrating an increase in glucocorticoids. This was achieved by the experiencing of environmental changes in the food protein and water level in the tadpole period, which, in turn, demonstrated a blunt glucocorticoid response when exposed to new stresses following metamorphosis [17]. These studies investigated how specific factors and responses in the early life history of the anuran species can influence the same factors and responses in their later life history.

The plastic responses may be more complicated due to the fact that, unlike other vertebrates, most anurans experience metamorphosis when maturing from tadpoles into adult frogs. Among the various plastic responses, the most important source of variability in tadpoles is the difference in the rate of metamorphosis with respect to environmental factors. The rate of metamorphosis can be changed by various environmental factors or stressors, such as the source of variability in water level, the protein composition of food, intra- or interspecific competition, the presence of predators, and chemical contamination (which, in turn, affects body size and health [9,18,19,20]). Individuals who experience a delayed metamorphosis period, with no body size increase, may have reduced opportunities in respect to growth, thereby negatively impacting reproduction or survival in their later life history [7,21,22,23,24]. Therefore, it has been suggested that there may be a mechanism by which delayed metamorphosis may have a compensatory effect on the terrestrial environment [7]. However, this has not been accurately determined. Several studies reported that the lasting effect changed the limb length and body width of a juvenile frog, as the metamorphosis period of a tadpole was accelerated or delayed [7,18,25,26]. Although these changes can have an impact in terms of the variation of anatomical traits, the quantification and comparative analysis of traits have rarely been studied [25].

When the environment that a tadpole has experienced causes changes in anatomical components related to locomotor performance after metamorphosis, it may affect the survival or dispersal in the life cycles of adult frogs. For example, a narrow pelvis, as well as an elongated ilium and urostyle, are anatomical components that can positively influence the jumping performance of frogs [27,28,29]. In addition, an increase in femur length entails a positive effect on swimming performance [27]. These locomotor traits can be the sole means for frogs without predation defense mechanisms to escape from the water to land, and from antipredator defenses. In addition, they can also result in a significant impact on the dispersal or migration behaviors within habitats, breeding grounds, and hibernation sites after metamorphosis.

We investigated whether the changes in locomotor performance, mediated by modifications in anatomical features post metamorphosis, are influenced by the presence of predators (specifically the dragonfly larva, *Anax parthenope*) that alter the metamorphosis period of the black-spotted pond frog (*Pelophylax nigromaculatus*). The dragonfly larva persists usually alongside the tadpoles of black-spotted pond frogs from South Korea; moreover, the dragonfly larva is a predator that directly consumes tadpoles. We assumed that juvenile frogs, who were subjected to predation pressure in their respective tadpole periods, would experience changes in their skeletal morphology, as well as changes in their locomotory performance after metamorphosis. As such, we identified the differences in metamorphosis and growth speed that took place under the influence of predation pressure, and quantified the tail shape of a tadpole, as well as the skeletal shape of a juvenile frog by utilizing landmark-based geometric morphometrics, in order to compare the morphology. The jumping and swimming speeds of frogs were also analyzed in order to determine the difference in locomotor performance.

## 2. Materials and Methods

### 2.1. Animal Collection and Experimental Condition

The black-spotted pond frog (*P. nigromaculatus*) is a semiaquatic pond frog, widely distributed in Korea, China, Japan, and far-eastern Russia [30]. This species can serve as an excellent model in which to assess plastic responses to the environment. This is because they are easy to sample and highly philopatric [30]. The tadpoles of these frogs inhabit various types of wetlands—such as water bodies, including river pools, channels, lakes, ponds, swamps, ditches, and rice paddy fields—and so their larvae can be exposed to various predators, such as aquatic insects or fish.

**Figure 1:**
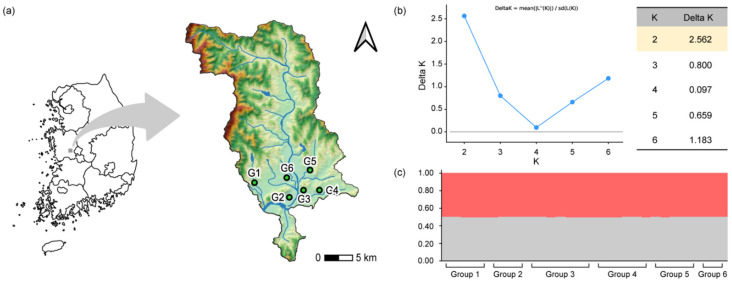
The sampling site of black-spotted pond frogs (*Pelophylax nigromaculatus*) and the genetic structure of the frogs. (**a**) Six sampling sites of black-spotted pond frog eggs (*Pelophylax nigromaculatus*). (**b**) The most suitable K value (K = 2 and delta K = 2.562) obtained using the delta K method in STRUCTURE Harvester. (**c**) The genetic structure of six groups when the delta K value was two. The genetic structure of six groups obtained using Bayesian clustering algorithms (STRUCTURE analysis) when the delta K value was two.

A total of 30 egg clutches were collected from six sites (G1 to G6); in addition, the number of masses per site was five (Figure 1a). It was found that there was no difference in respect of the genetic structure in this population [31]. However, we conducted a genetic analysis on this issue in order to confirm this with certainty. An analysis of population genetic structure was conducted in order to exclude whether the plasticity was affected by a population genetic factor. From each site, three to seven adult frogs were collected, and genomic DNA was extracted in the toe samples. Freezing subjects with ice-cold water can be used as an anesthesia for ectothermic vertebrates, such as amphibians and reptiles [32]. We collected the third toe on the right hind leg from the frogs that were anesthetized in ice-cold water. This was conducted in order to avoid any unnecessary pain in respect to the frogs.

The genome DNA of the toe samples was extracted using DNeasy Blood and Tissue kits (Qiagen, Hilden, Germany), according to the manufacturer’s protocol. In total, seven primers (Rnh-1, Rnh-2, Rnh-3, Rnh-4, Rnh-6, Rnh-10, and Rnh-12) were used in order to amplify the microsatellite loci of the frogs, according to previously reported procedures [33]. In addition, amplified loci were visualized using a Seq-Studio Genetic Analyzer (Thermo Fisher-Applied Bio systems, Foster City, CA, USA). A STRUCTURE version 2.3.4. [34] was used for the purposes of Bayesian clustering. This analysis allows one to determine the genetic structure of individuals *i* by the process of confirming that certain genetic material is derived from a common ancestor *K*. Each analysis consisted of 100,000 simulations, following the burn-in of 100,000 simulations. The STRUCTURE Harvester [35] was used for the delta K method [36] for the purposes of the identification of the most appropriate K value. The three independent runs in each range of one to six possible clusters were used for the STRUCTURE Harvester. Moreover, in the STRUCTURE Harvester, the best-supported K value was determined to be 2 (Figure 1b). In the STRUCTURE analysis, the frog populations at six sites possessed no population genetic differences; furthermore, they were also identified as one population (Figure 1c).

The frog egg clutches were transported to the laboratory in plastic boxes. They were then raised in clear water for 15 days. Tadpoles from the five egg clutches in each collection site were raised together in a single container. After hatching, we randomly extracted 60 tadpoles per collection site that had hatched on the same day; further, they were kept in separate cages. A total of 360 tadpoles were used in the experiment. Until the end of the experiment, all tadpoles were bred in a container (460 mm × 300 mm × 170 mm) with 10 L of water and air generators that operated under a 12 h/12 h light/dark regime, with 3 W LED light; lastly, the air temperature was maintained at 23 ± 1 °C. Dog food (Original dog, Origen, Edmonton, AB, Canada) was used to feed the tadpoles, and an amount equivalent to about 3% of the tadpole weight was supplied every 3 days. Leftover food was removed the next day and the water was only half changed once a week. The water level was checked daily and kept constant until the end of the experiment. When we found that the tail of the tadpoles was absorbed (i.e., Gosner stage 45), we placed a floating piece of plastic in each container. The experimental procedures and animal maintenance were performed in accordance with the regulations and approval of the Experimental Animal Ethics Committee of Kongju National University (KNU_2019-01).

When the external gills of hatchlings disappeared and the internal gills of tadpoles began to appear (i.e., Gosner stage 26), 60 tadpoles were divided into two groups of 30 individuals each (a set); furthermore, one group was indirectly (i.e., through visual and chemical cues) exposed to the predator (predation group), whereas the other group was not exposed to the predator (control group). A total of six sets were made, four sets of which were used for the experiment, and two sets were left as spare sets for the purposes of supplement when tadpoles died in the experiment sets.

The plastic container had a separation compartment that contained the dragonfly larva (*Anax parthenope*). The tadpoles were separated from the dragonfly larva area by a segregation compartment. However, this was a segregation compartment that still allowed the flow of chemical and visual cues by the dragonfly larva. Further, the dragonfly larvae were fed separately reared tadpoles of the same species, supplied every other day. In order to minimize the spatial impact, a separate compartment (where there were no predators) was also placed in the container of the control group.

We collected five tadpoles from each container in order to confirm that there was no difference in the length and weight of tadpoles between the predation group and control group at the start of the experiment (Gosner stage 26). We tapped the collected tadpoles with a piece of gauze in order to remove moisture. The side of the tadpole was photographed for the purposes of length measurement. The body length of the collected tadpoles was measured from snout to tail tip by using the ImageJ software [37]. The weights of the collected tadpoles were measured using a digital balance. Length and weight were measured to the nearest 0.001 units. A total of 20 tadpoles per treatment group, between both the predation group and control group, were used for the comparison of initial body length and weight, whilst utilizing an unpaired *t*-test.

### 2.2. Analysis of Growth and Developmental Rate

We assessed the developmental condition of the tadpoles daily, removed the tadpoles that reached Gosner stage 39, photographed the side of the tadpole by following the same methodology described, and measured their weights. The collected tadpoles were removed from the containers, and the length, weight, and time to reach Gosner stage 39 were calculated in order to determine the growth status and development rate of the tadpoles. Five tadpoles (Gosner stage 39) were collected from each container (20 tadpoles were extracted from each group, i.e., both treatment and control). Then, we analyzed for side photos, length, and the weight of collected tadpoles in the same process as mentioned above. Further, it must be noted that the analyzed tadpoles were not returned to the container.

**Figure 2:**
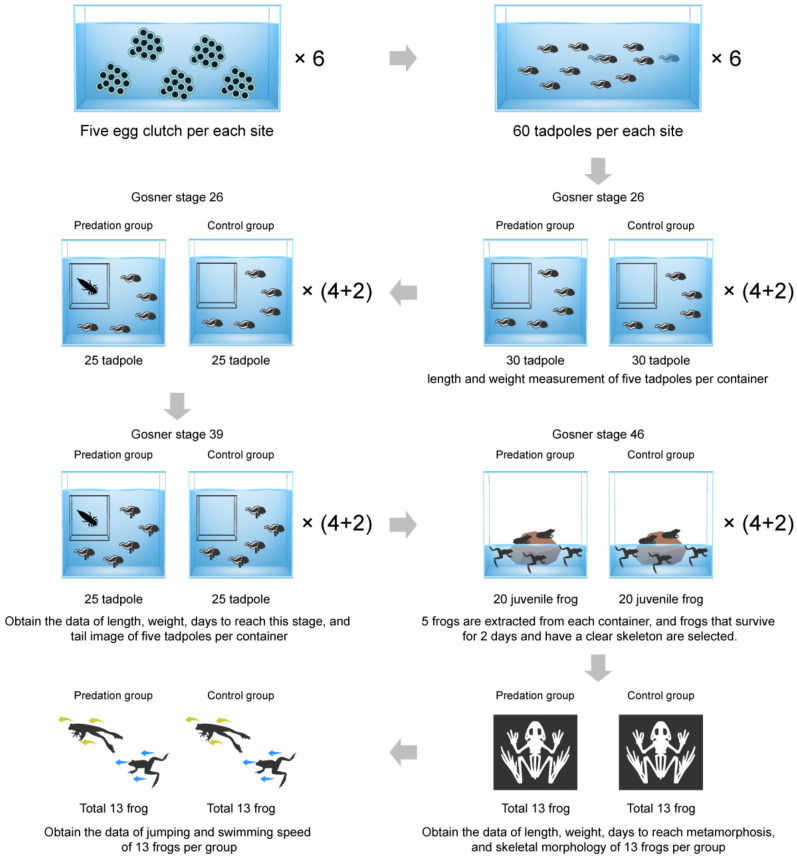
Flow chart for the process of this experiment. Experiments started at the Gosner stage 26 in respect to the tadpole. Individual traits were measured at Gosner stage 39 (tadpoles) and Gosner stage 46 (juvenile frogs). Of the total six experimental sets, four sets were used for experiments and two sets were kept as reserve sets, for the purposes of replenishment when tadpoles died.

In addition to the above, we measured the length, weight, and the rate of metamorphosis regarding the juvenile frogs after metamorphosis. Five juvenile frogs (Gosner stage 46) were collected in each container (i.e., 20 juvenile frogs from each group). The juvenile frogs were removed under the following conditions: frogs that died if maintained for two days after metamorphosis; frogs that did not exhibit a clear skeletal shape on an X-ray film. Thus, 13 juvenile frogs per group were used for the purposes of data analysis. The length of the juvenile frogs was measured according to a snout–vent length, to the nearest 0.01 mm, by using a digital caliper. In addition, the weight of the frogs was measured, to the nearest 0.001 g, by utilizing a digital balance. After the length and weight measurements, the juvenile frogs were transferred to separate containers (460 mm × 300 mm × 170 mm), which contained both land and clear water. Furthermore, these containers were maintained for 48 h and then used in experiments in order to compare locomotor performance. We then summarized the process of this experiment graphically (Figure 2).

### 2.3. Measurement of Jumping and Swimming Speed

We prepared two glass tanks (600 mm × 500 mm × 450 mm) for the purposes of measuring the jumping and swimming performances. One glass tank was used in order to observe the jumping of the frogs. Further, the other glass tank was used to observe the swimming of the frogs. The two glass tanks were all covered with white paper, except for the top. Furthermore, a styrofoam land was fixed on one side in a glass tank that was designed to observe swimming. The water was filled to a water level of about 10 cm. The lighting was manufactured using 3 W LED light. A digital camera (iPhone 11 Pro, Apple, Los Altos, CA, USA) was used to film the jumping and swimming of the frogs from a top-down view. As the efficiency or maximum ability of locomotory performance may vary depending on temperature [38], the experiment was performed at a room temperature of 23 °C.

In the jumping video, the frogs commenced jumping from one side of the tank. Via utilizing a thin paint brush, we tapped the urostyle of the frogs in order to induce a strong jump. The experiment was repeated three times for each individual, and there was a break of 5 min among each experiment. In the case of consecutive jumps in one experiment, the jumping action with the highest speed was selected. In the swimming video shoot, the frog was placed on styrofoam land at the end of a glass tank. After that, a thin paint brush was used to allow the individual to enter the water from land. Swimming behavior immediately after drowning was excluded, and only the subsequent swimming behavior was used for the purposes of analysis. Similarly, three swimming experiments were repeated and a 5 min break between each experiment was implemented. In the case of a consecutive swim in one experiment, the swimming action with the highest speed was selected.

We split the video into 30 frames per second and performed the analysis using ImageJ [37]. First, we measured the distance that was traveled by the frog by them jumping once or swimming once. The distance at which the frog jumped and landed was measured based on the tip of the frog’s snout. Furthermore, the jumping start of the frog was considered when the tip of the snout moved, and the end of the jump was considered when the tip of the snout stopped moving. Swimming speed was also measured, and the starting point of the swimming distance was considered as the retracted state before the frog opened its legs for swimming. In addition, the ending point of the swimming distance was considered as returning to the retracted state before the frog opened its legs again after swimming. Then, by measuring the time to reach this distance, the jumping and swimming speed (distance/time) were calculated. We calculated the jumping and swimming speeds of each individual by utilizing the average value of the data extracted from a total of three experiments.

### 2.4. Identification of Morphological Change

**Figure 3:**
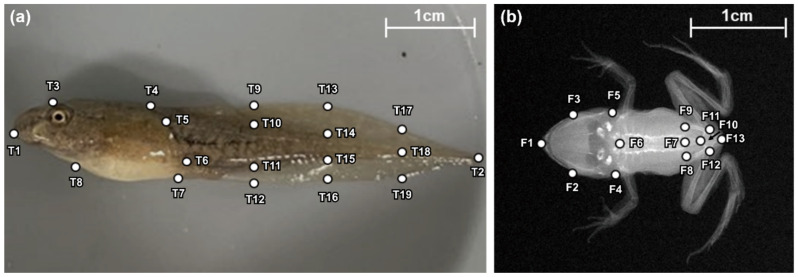
Left-side image of a tadpole, as well as the X-ray bone image of a juvenile frog. (**a**) Nineteen landmark points were designated to represent the body and tail shapes of the tadpole, as well as to compare the morphological difference between the predation and control groups. (**b**) Thirteen landmark points were assigned in order to quantify and compare the skeletal morphology of the head and lower body in juvenile frogs.

Landmark-based geometric morphometrics were used to analyze the morphological changes in tadpoles and juvenile frogs. The left-side image of the tadpole was used in order to analyze the shape of the tadpole tail. Furthermore, the skeletal shape of juvenile frogs was analyzed in order to identify the shape of the skull, pelvis, ilium, and urostyle. Further, 2D skeleton images were captured from the dorsal direction by using an X-ray imaging system (EMT-F70, Softex, Tokyo, Japan). We then digitized the landmark points in respect to the shape of the tadpole tail by using the TpsDig software [39]. Nineteen landmarks that were chosen as representative of the body and tail shapes were designated in the tadpoles (Figure 3a). Thirteen landmarks representing the skeletal shape of the head and lower body were designated as the juvenile frogs (Figure 3b). Furthermore, we used the MorphoJ software (version 1.07a, Manchester, UK) in order to convert digitized landmark coordinates into Procrustes coordinates. Next, a canonical variate analysis (CVA) was employed in order to compare the morphological differences between the predation and control groups, as well as to calculate the contribution (%) of each canonical variate (CV) axis in the CVA. The variation in tail shape between the four treatment groups was visualized using a rectangular grid and a wireframe graph of CV1. Additionally, we compared the skeletal lengths of the forelimb (i.e., the sum of the length of the humerus and radio–ulna); hindlimb (sum of the length of the femur and the tibia–fibula); and the urostyle, which are skeletal traits that are related to locomotor performance in frogs. The ImageJ software [37] was used in order to measure the bone length of the experimental subjects to the nearest 0.001 mm.

### 2.5. Statistical Analysis

An unpaired *t*-test was used in order to compare the length and weight in both the tadpoles and juvenile frogs. The developmental rate of the tadpoles and the metamorphosis timing of the juvenile frogs were compared by utilizing an unpaired *t*-test. We also analyzed the difference in respect to the length of the bones in the limbs and urostyle of the subjects. In addition, the jumping speed and the swimming speed of juvenile frogs between the predation group and control group was analyzed by an unpaired *t*-test. The assumptions of the unpaired *t*-test were confirmed by a Levene’s equal variances test and the Shapiro–Wilk normality test. Statistical differences were significant at *p* < 0.05. In respect to this, GraphPad Prism version 8.0 for Windows (GraphPad Software, San Diego, CA, USA) was used in order to perform the statistical analyses.

## 3. Results

### 3.1. Differences in the Physical Conditions, Development, and Tail Shape of Tadpoles as Caused by Predator Pressure

The initial (Gosner stage 26) length (t _(38)_ = 0.755, *p* > 0.05) and weight (t _(38)_ = 0.049, *p* > 0.05) of the tadpoles did not appear to have resulted in a difference between the predation group (length = 14.26 ± 1.46 mm, weight = 0.02 ± 0.01 g) or the control group (length = 14.38 ± 1.39 mm, weight = 0.02 ± 0.01 g). In respect to this, it must be noted that the tadpoles that were used for length and weight measurements were excluded from the experiment.

**Figure 4:**
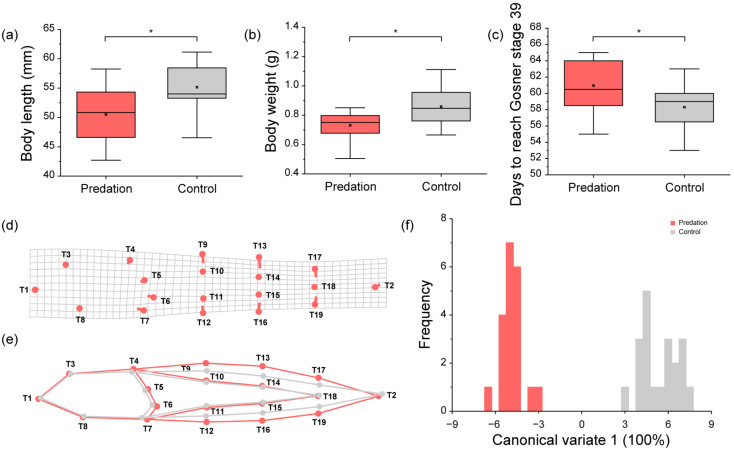
Comparison of the (**a**) body length; (**b**) body weight; (**c**) development rate; and (**d**–**f**) the body and tail shape in tadpoles (Gosner stage 39) between the predation and control groups: (**d**) The rectangular grid with landmark points and vectors of the canonical variate 1 (CV1); (**e**) the wireframe graph of the tadpole in respect to the predation and control groups from CV1; and (**f**) the distribution of the CV1 value in the tadpoles between the predation and control groups. The red and gray boxes represent the tadpoles from the predation and control groups, respectively. The box plots detail: the mean (central square dot); median (central band); 25th and 75th percentiles (**bottom** and **top** of boxes); and range within a 1.5 interquartile (the **bottom** and **top** of the line). Significant differences (*p* < 0.05) were determined using an unpaired *t*-test and are represented with an asterisk (*). Circles in the deformation grid, as well as the grey points and lines in the wireframe graph, indicate the body and tail shape of the individuals with the lowest CV value. The lines in the deformation grid, as well as the red points and lines in the wireframe graph, indicate the change in body and tail shape via an increase in CV value.

The control tadpoles (length = 55.15 ± 4.05 mm, weight = 0.86 ± 0.13 g) that were in Gosner stage 39 possessed a higher body length (t _(38)_ = 3.285, *p* = 0.002) and weight (t _(38)_ = 3.664, *p* = 0.001) than the tadpoles of the predation group (length = 50.50 ± 4.87 mm, weight = 0.73 ± 0.09 g) who were in Gosner stage 39 (Figure 4a,b). However, the time that was incurred in respect to the tadpoles in the predation group (time = 60.95 ± 3.36 days) to reach Gosner stage 39 was longer (t _(38)_ = 2.781, *p* = 0.008) than the time in which the control group (time = 58.30 ± 2.62 days) was able to reach Gosner stage 39 (Figure 4c).

Canonical variate analysis (CVA) was used in order to analyze the body and tail shape of the tadpoles (Gosner stage 39) between both the predation and control groups. The canonical variate 1 (CV1) axis explained the 100% morphological variation of the bodies and tails of the tadpoles. The higher the CV1 was represented, the smaller the body became; in addition, the tail also became longer and the caudal fin narrower (Figure 4d,e). The tadpoles in the predation group possessed the lower CV1 values than the tadpoles in the control group (Figure 4f). As such, this means that the tadpoles in the predation group possessed larger bodies, shorter tails, and deeper caudal fins than the tadpoles in the control group.

### 3.2. Comparison Regarding the Physical Conditions, Metamorphosis Timing, and Skeletal Shape of Juvenile Frogs

**Figure 5:**
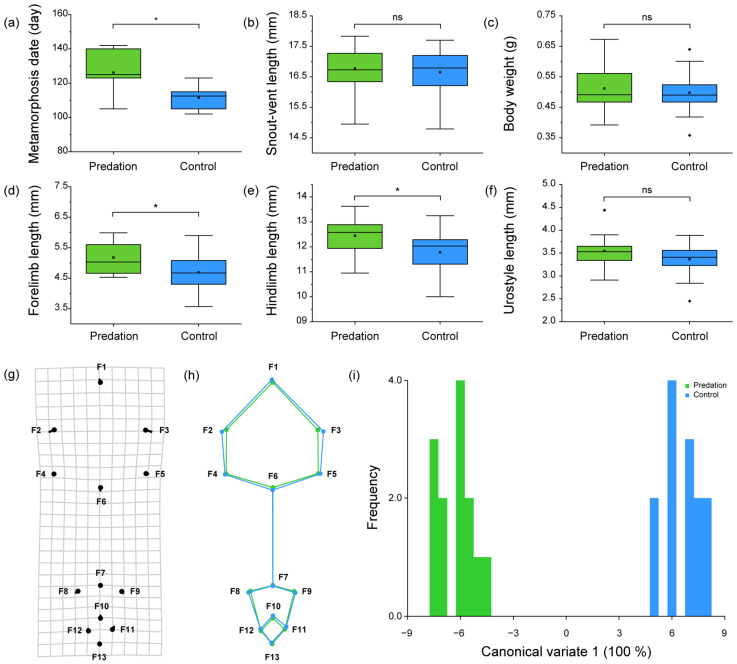
Comparison of the: (**a**) metamorphosis rate; (**b**) snout–vent length; (**c**) body weight; (**d**) forelimb (i.e., the humerus and radio–ulna) length; (**e**) hind limb (i.e., the femur and tibio–fibula) length; (**f**) urostyle length; and (**g**–**i**) the skeletal shape of the head and lower body of the juvenile frogs (Gosner stage 46) between the predation and control groups; (**g**) the rectangular grid with landmark points and vectors of the canonical variate 1 (CV1); (**h**) the wireframe graph of juvenile frogs in the predation and control groups in respect to the CV1; and (**i**) the distribution of the CV1 value in juvenile frogs between the predation and control groups. The green and blue boxes represent the tadpoles from the predation and control groups, respectively. The box plots show the: mean (central square dot); median (central band); 25th and 75th percentiles (the **bottom** and **top** of the boxes); range within a 1.5 interquartile (the **bottom** and **top** of the line); and outlier (the diamond dots). Significant differences (*p* < 0.05) were determined using an unpaired *t*-test and are represented with an asterisk (*). Circles in the deformation grid, as well as the green points and lines in the wireframe graph indicate the skeletal shape of the individuals with the lowest CV value. Sticks in the deformation grid, as well as the blue points and lines in the wireframe graph, indicate the change in skeletal shape by an increase in CV value.

The period of time to metamorphosis (Gosner stage 46) in the predation group (time = 126.15 ± 11.90 days) in respect to the juvenile frog was longer (t _(24)_ = 4.378, *p* < 0.001) than the time that was required in the control group (time = 109.61 ± 4.98 days) (Figure 5a). Moreover, the body length (t _(24)_ = 0.399, *p* = 0.693) and weight (t _(24)_ = 0.466, *p* = 0.646) were not significantly different between the predation group (length = 16.77 ± 0.81 mm, weight = 0.51 ± 0.08 g) and the control group (length = 16.65 ± 0.76 mm, weight = 0.50 ± 0.07 g) in Gosner stage 46 (Figure 5b,c).

The skeletal length of the forelimb (t _(24)_ = 2.073, *p* = 0.049) that combines the humerus, radio–ulna, and the hindlimb (t _(24)_ = 2.077, *p* = 0.049), as well as that which combines the femur and tibio–fibula, was longer in the frogs of the predation group (forelimb = 5.18 ± 0.51 mm, hindlimb = 12.45 ± 0.79 mm) than in the frogs of the control group (forelimb = 4.69 ± 0.66 mm, hindlimb = 11.78 ± 0.85 mm) in Gosner stage 46 (Figure 5d,e). Contrarily, the length of the urostyle did not differ (t _(24)_ = 1.251, *p* = 0.223) between the predation group (3.55 ± 0.43 mm) and the control group (3.37 ± 0.45 mm) in Gosner stage 46 (Figure 5f).

The CV1 axis can help explain the 100% morphological variation in respect to the skeletal shape, regarding the head and lower body of the juvenile frogs (Gosner stage 46). The higher the CV1 was represented, the more the skull widened; likewise, the higher the CV1, the pelvis also was noted to have slightly widened (Figure 5g,h). The juvenile frogs in the predation group possessed lower CV1 values than the frogs in the control group (Figure 5i). This, thus, represents the fact that the juvenile frogs in the predation group possessed a narrower skull than the frogs in the control group. However, it must be noted that there was little change in the shape of the pelvis and ilium.

### 3.3. Comparison of Jumping and Swimming Speeds between Predation Group and Control Group

**Figure 6:**
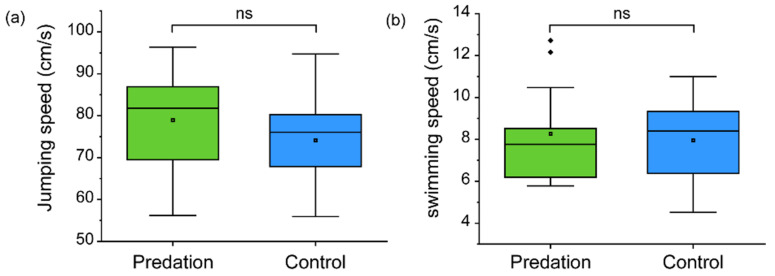
Comparison of the (**a**) jumping and (**b**) swimming speeds in juvenile frogs (Gosner stage 46) between the predation and control groups. The green and blue boxes represent the tadpoles from the predation and control groups, respectively. The box plots show the: mean (central square dot); median (central band); 25th and 75th percentiles (the **bottom** and **top** of the boxes); range within the 1.5 interquartile (the **bottom** and **top** of the line); and outlier (diamond dots).

After metamorphosis from tadpole to juvenile frog, the jumping speed was not significantly different (t _(24)_ = 1.075, *p* = 0.293) between the predation group (78.92 ± 11.03 cm/s) and the control group (74.11 ± 11.78 cm/s) in Gosner stage 46 (Figure 6a). Similarly, the swimming speed did not differ (t _(24)_ = 0.383, *p* = 0.705) between the predation group (8.26 ± 2.25 cm/s) and the control group (7.95 ± 1.91 cm/s) in Gosner stage 46 (Figure 6b)

## 4. Discussion

**Figure 7:**
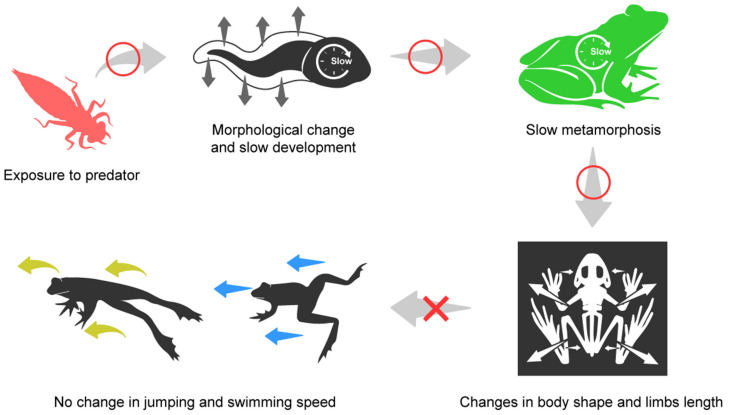
The response of tadpoles to the presence of predators and the effects of such a response following metamorphosis. The shape of the tail and the rate of metamorphosis in tadpoles, as well as—subsequently—the skeletal shape in juvenile frogs were all altered in the presence of a predator. However, there was no change found in respect to the jumping and swimming speeds of these subjects in the presence of predators.

In this study, the developmental and morphological response of tadpoles according to the presence of predators was confirmed, as well as whether this could lead to anatomical and behavioral changes in juvenile frogs (Figure 7). Prior to interpreting our results, there were many disparate results in previous studies regarding tadpole responses. This type of disparity has been defined as context dependence. Furthermore, solving this is one of the critical challenges to be addressed in order to improve the understanding and prediction of ecology [40]. Therefore, we tried to organize the contents based on the disparate results, and thus interpret our results better.

Through conducting this study, it was found that predation pressure slowed the development of tadpoles. In addition, predation pressure also induced a smaller body size and weight in the same life-history stage for the affected tadpole. In addition, the caudal fins of tadpoles under predation pressure became wider. In respect to this, changes in the tail of tadpoles via non-consumption effects were previously reported [11,41]; further, these studies show that tadpoles who possess a change in the tail can escape predators more reliably and successfully [42,43,44,45]. Additionally, smaller body size and slower development were demonstrated to be related in certain previous studies [6,42,46,47]. The delay of development that is induced by predation pressure may be a result of the interaction between the behavioral changes and resource availability that takes place due to the risk of predation [48], the decrease in the competitive ability [7,8], and the lowering of the trophic level [46]. All of this can result in delays in respect to the growth of tadpoles and their metamorphosis.

However, there are also contradictory results. Certain studies show faster metamorphosis timing when the subjects were exposed to predators [26,49]. Furthermore, predation risk acts as a stressor, thereby altering the endocrine system and triggering the release of the glucocorticoid hormone corticosterone [12]. Corticosterone may accelerate metamorphosis through a synergistic effect that increases tissue sensitivity to thyroid hormones [50,51]. The difference in results in that type of tadpole metamorphosis is accelerated or delayed according to predation pressure, and is suggested to be due to the differences within the species. More specifically, the length of the tadpole period differs among species [52]. Species with long larval periods (i.e., an average of 69–96 days) demonstrate slower metamorphoses when there is predator exposure [53,54], whereas species with a shorter tadpole period (an average of 10–40 days) show an accelerated metamorphosis [55,56]. In our study, it is possible that the slow development rate due to predation pressure is due to the relatively long tadpole period of this species. The metamorphosis timing of juvenile frogs in our study was especially slower, at around 100 to 120 days when compared to the 48 to 75 days that was evidenced in previous studies [57,58,59,60] that analyzed the same species. This may be due to the protein content of the food, or the laboratory temperature conditions, but such an explanation cannot be accurately confirmed from the papers of studies on the same species. Indeed, the slow development appears to be due to complex competition and food interactions, due to low growth conditions and the presence of predators.

We analyzed the skeletal morphology in order to confirm anatomical changes in juvenile frogs that experienced predation pressure. In particular, skeletal traits related to jumping performances were analyzed in detail. Juvenile frogs under predation pressure possessed a narrower skull and longer limbs during the tadpole period. However, the shape of the pelvis, ilium, and urostyle was almost unchanged. Many studies have reported morphological changes due to predation pressure, but the results were heterogeneous; among these studies, a collection even reported morphological changes through delayed metamorphosis, whereas other studies reported morphological changes through accelerated metamorphosis [6,26]. Nevertheless, the changes in body width and limb length according to the delay in the acceleration of metamorphosis were consistent. When the metamorphosis period was delayed, the body width decreased and the limbs became longer [6], whereas when the metamorphosis period was accelerated, the body width became increased, and the limbs became shorter [26]. This result was suggested as a difference in thyroid hormone exposure due to the delay or acceleration of metamorphosis, rather than due to the direct response to predation pressure [61]. In certain studies, this suggestion was judged to be consistent by confirming similar morphological changes according to the extension of the metamorphosis period under such conditions found in dry ponds, low protein in respect to food, or low temperature [4,18,25]. Our results support the notion that these changes were influenced by the delayed metamorphosis rather than by a predator-specific response. Therefore, similar changes are expected in other related factors that may alter the metamorphosis period.

Although there was no difference in the shape of the pelvis, or in the length of the ilium and urostyle that could affect the jumping performance, it was expected that the fine longer limb length could increase the two types of locomotory performances (i.e., jumping or swimming). However, our expectations were wrong. There was no difference in the jumping and swimming speed between the two groups. Indeed, certain researchers have also studied the change in locomotory performance via the change in metamorphosis periods in tadpoles, but the results were different. Tadpoles grown at a low density were larger in body size and demonstrated a further jumping performance than tadpoles at a high density; having said this, however, there was no difference found in the hind limb length [62]. Tadpoles exposed to a decrease in water levels exhibited accelerated metamorphosis and decreased jumping ability due to their small body size and short hind legs [4]. In addition, although subadult frogs who were exposed to conditions of low temperature and low protein composition in food showed delayed metamorphosis and longer legs, the results of normalizing the jumping performance to the relative leg length showed no difference in respect to the motor performance [25]. In the case of aquatic frogs, the metamorphosis of tadpoles accelerated according to the degree of predation pressure. In addition, smaller juvenile frogs emerged, but there was no difference in their swimming performance as standardized by body length [56]. Among the studies that possessed differences in body size after metamorphosis, studies that did not standardize body size, or studies that simply calculated jumping performance in terms of distance, found a significant difference in locomotory performance. However, it was difficult to find a difference in studies that did not. Similarly, in our results, there was no difference in the length and weight of the frogs after metamorphosis; furthermore, there was no difference in the jumping and swimming speed either.

Previous researchers have predicted that the intensity of change in the hind limbs is likely to be too weak to affect functional traits [6,26,61]. In order to engender a significant difference in jumping performance, a difference in hind leg length of about 10% should appear [29]. In our results, the difference in hindlimb length was extremely small (i.e., the difference average was 4.4%). Moreover, there was almost no difference in the width of the pelvis and the lengths of the ilium and urostyle. It appears, then, that these subtle morphological changes were not sufficient to significantly change locomotory performance. Phenotypic plasticity via delayed metamorphosis may be a by-product of altered tadpole periods, rather than an adaptive response toward predator escape ability or for terrestrial life history [6]. Through this study, we also identified anatomical traits. Except for the length of limbs, there was no significant difference found in respect to the anatomical traits that could affect the locomotory performance. As the forelimb becomes shorter in frogs, the center of gravity moves to the rear, thereby helping increase acceleration when jumping. As such, the hindlimb becomes longer in these frogs and the jumping performance increases as a result [29,63]. Having said this, simultaneous changes in both components occurred in our study. There may not be a significant difference in terms of jumping due to the fact that the increase and decrease in locomotor performance occurred at the same. Similarly, in respect to jumping performance, the length of the hind legs—especially the length of the femur—indicates an increase in the swimming speed [27]; however, having said this, we also found that there was no difference in our results. It has been suggested that these morphological changes due to a delay or acceleration in metamorphosis should be confirmed due to the fact that they can play a positive role in the life history of frogs following metamorphosis [6]. However, our results did not find any benefit in either aquatic nor terrestrial environments. Indeed, perhaps it is likely that the morphological changes will not entail a critical influence on the life history of frogs following metamorphosis. Strictly speaking, our results show that there were no changes in the anatomical traits that could lead to differences in jumping and swimming performance. Phenotypic plasticity appears to change a species into a more suitable form in response to changes in the environment of their respective habitats, but plasticity does not necessarily appear to be an adaptive response or a positive trait.

## 5. Conclusions

In spite of targeting the same factors in this study, the results from several previous studies, including ours, were concluded with disparate results in terms of different traits among the anuran species, different developmental stages that are exposed to certain factors, and the strength of the factors whereby the subjects responded to environmental pressures. However, the responses to changes in body size, body weight, and morphology according to the rate of development, appears to be similar. Even frogs that have experienced delayed or accelerated metamorphosis, including predation pressure, will not find an increase in locomotory performance due to the subtle changes in limb length and body shape, unless there is also a change in body size. Although altered morphology may have an ecological role that we have not thus far detected, our results still support the notion that delayed metamorphosis in tadpoles may be detrimental to their later life-history.

## Data Availability

Not applicable.
